# Gut microbiota-derived metabolites target C5AR1/KDM2A/HCAR3 axis in inflammatory bowel disease: a multi-machine learning algorithms and molecular docking study

**DOI:** 10.3389/fcimb.2026.1912373

**Published:** 2026-08-21

**Authors:** Shanshan Hu, Lin Fu, Jing Gao, Qiongya Guo, Shuangyin Han

**Affiliations:** Department of Gastroenterology, Henan Provincial People’s Hospital/People’s Hospital of Zhengzhou University/People’s Hospital of Henan University, Zhengzhou, Henan, China

**Keywords:** C5aR1, gut microbiota, HCAR3, inflammatory bowel disease, KDM2A, machine learning, molecular docking

## Abstract

**Background:**

Inflammatory bowel disease (IBD) is a chronic recurrent disorder. Gut microbiota-derived metabolites regulate intestinal homeostasis, but their molecular mechanisms in IBD remain unclear. Current studies lack systematic “microbiota-metabolite-target” network mining with multi-method validation. This study integrates network pharmacology, three machine learning algorithms, and molecular docking to construct this regulatory network in IBD.

**Methods:**

Transcriptome data were obtained from the Gene Expression Omnibus (GEO) database. Differentially expressed genes (DEGs) were identified using limma (p < 0.05, |log_2_FC| > 0.5). Weighted gene co-expression network analysis (WGCNA) with an optimal soft threshold of β = 7 was performed to identify key module genes. Candidate genes were obtained by intersecting DEGs, gut microbiota-associated genes from the gutMGene database, and WGCNA module genes. Gene Ontology (GO) and Kyoto Encyclopedia of Genes and Genomes (KEGG) enrichment analyses were conducted to explore the functional roles of candidate genes. Core genes were identified using three machine learning algorithms (LASSO, Boruta, and SVM-RFE), followed by protein-protein interaction (PPI) network analysis. Molecular docking was performed to assess the binding affinities between hub proteins and gut microbiota-derived metabolites.

**Results:**

A total of 885 DEGs were identified between the IBD and control groups, including 463 upregulated and 422 downregulated genes. WGCNA identified 280 key module genes from the purple and yellow modules. The intersection of DEGs, gut microbiota-associated genes, and WGCNA module genes yielded 19 core candidate genes. PPI network analysis combined with three machine learning algorithms jointly identified C5AR1, KDM2A, and HCAR3 as core hub genes. ROC curve analysis demonstrated that all three hub genes achieved AUC values greater than 0.7 in both the training and validation sets, indicating excellent diagnostic performance for IBD. Enrichment analysis revealed significant associations with the TNF, NF-κB, and IL-17 signaling pathways. Molecular docking confirmed stable binding of C5AR1 with 1,3-Diphenylpropan-2-Ol (-7.87 ± 0.83 kcal·mol^-^¹) and HCAR3 with 3-Indolepropionic Acid (-6.35 ± 0.70 kcal·mol^-^¹), both below −5.0 kcal·mol^-^¹.

**Conclusion:**

This study first constructs a “gut microbiota-metabolite-hub gene” axis in IBD, providing a computational framework for microbiota-targeted precision therapy, and identifying C5AR1/KDM2A/HCAR3 as computationally predicted diagnostic biomarkers and 1,3-Diphenylpropan-2-Ol/3-Indolepropionic Acid as candidate intervention molecules that warrant further experimental validation.

## 1 Introduction

Inflammatory bowel disease (IBD) is a group of idiopathic digestive disorders characterized by chronic recurrent intestinal inflammation, primarily including ulcerative colitis (UC) and Crohn’s disease (CD) (Tavakoli et al., 2021; Bruner et al., 2023; Ding et al., 2025). The global incidence of IBD is rising annually, particularly in developing countries (Hracs et al., 2025) and China, where increasing age-standardized rates of incidence and mortality severely impair patients’ health and quality of life (Yu et al., 2024; Chen et al., 2026). IBD presents with distinct features: UC typically involves diarrhea, bloody mucoid stools, and post-defecation abdominal pain (Wangchuk et al., 2024), while CD manifests as intermittent abdominal pain, pasty stools, fistulas, and perianal lesions (Singh et al., 2024; Cicerone and Kotze, 2025). IBD follows a chronic relapsing course, with persistent mucosal inflammation leading to serious complications (e.g., bleeding, perforation, PSC, thromboembolism) and increased CRC risk after 10 years (Faggiani et al., 2024; Kapadia et al., 2025). Current treatments (e.g., aminosalicylates, corticosteroids, surgery) aim to control inflammation but are limited by variable efficacy, notable side effects, high relapse rates, and lack of cure (Cai et al., 2021; Yang et al., 2026). Therefore, identifying key regulatory nodes, diagnostic biomarkers, and therapeutic targets is urgently needed to optimize IBD management and improve patient outcomes.

The gut microbiota, known as the human “second genome,” maintains homeostasis closely linked to intestinal health, and its dysbiosis is a core pathogenic mechanism of IBD (Xie et al., 2026). Gut microbiota-derived metabolites, which are closely associated with host genes (as documented in the gutMGene v2.0 database, a comprehensive resource for gut microbe-metabolite-host gene associations), regulate intestinal barrier function, immune responses, and inflammation (Chulenbayeva et al., 2025; Kim et al., 2025). IBD patients exhibit significant gut microbiota dysbiosis, characterized by enrichment of pro-inflammatory genera (e.g., g_Fusobacterium, g_Morganella) and depletion of SCFA-producing bacteria (e.g., g_Ruminococcus, g_Agathobacter) (Ding et al., 2025). However, the specific molecular mechanisms remain incompletely understood. Current studies predominantly use single methodologies. For example, most previous studies only focused on single targets or pathways via network pharmacology, without cross-validation by multiple machine learning algorithms or verification of molecular interactions between metabolites and targets (Yao et al., 2025) This hinders the elucidation of core regulatory patterns and limits deeper understanding and translational applications in IBD pathogenesis.

This study aims to elucidate the molecular mechanisms by which gut microbiota-derived metabolites regulate IBD through the “microbiota-metabolite-target” axis. This will fill the gap in the systematic regulatory network of IBD and provide new targets for precision therapy, aligning with the clinical demand for effective IBD intervention. The specific objectives are as follows: 1) Identify IBD-related DEGs from Gene Expression Omnibus (GEO) transcriptome data and screen candidate genes by intersecting differentially expressed genes (DEGs), gut microbiota-associated genes (from gutMGene) and weighted gene co-expression network analysis (WGCNA) key module genes; 2) Screen core hub genes via three machine learning algorithms and PPI network analysis, and validate their diagnostic value; 3) Evaluate the binding affinity between hub proteins and gut microbiota-derived metabolites by molecular docking, and construct the “gut microbiota-metabolite-target” regulatory axis.

## 2 Methods

### 2.1 Data acquisition and preprocessing

The transcriptome sequencing data used in this study were obtained from two IBD-related datasets in the NCBI GEO database (https://www.ncbi.nlm.nih.gov/geo/). The GSE3365 dataset, generated on the Affymetrix GPL96 platform, served as the training set and included samples from 85 IBD patients (case group) and 42 healthy controls (control group). The GSE47908 dataset, based on the GPL570 platform, was used as the validation set for core target gene expression, comprising 45 IBD patients and 15 healthy controls.

To address potential platform-specific biases between GPL96 and GPL570, we performed ComBat batch-effect correction using the sva package. Prior to this, we carried out probe-to-gene annotation: for GPL96, we used hgu133a.db; for GPL570, hgu133plus2.db. Probes without gene annotation or gene symbols were discarded; for genes with multiple probes, expression values were collapsed by computing the mean per gene. This annotation and collapsing step was performed after RMA normalization and before ComBat. As shown in **Supplementary Figure 1A**, raw expression of C5AR1, HCAR3, and KDM2A showed clear batch-specific deviations between platforms; after ComBat (**Supplementary Figure 1B**), medians and interquartile ranges were well aligned, confirming effective removal of platform-related distribution shifts.

### 2.2 Identification of differential genes and correlation analysis

Differential expression analysis between the IBD group and the healthy control group was performed using the R package ‘limma’ (version 3.60.4). Statistical significance was defined as p-value < 0.05 and |log_2_FC| > 0.5, and genes not meeting these criteria were excluded from further analysis. To visualize the overall distribution of DEGs, a volcano plot was generated using the ggplot2 package (version 3.5.1), with the top five up- and down-regulated genes explicitly labeled. Additionally, a heatmap was created using the ComplexHeatmap package (version 2.20.0) to illustrate the expression patterns of the top ten most significantly up- and down-regulated genes across all samples, where red represents the IBD group and blue represents the healthy control group.

### 2.3 WGCNA analysis identifies key module genes

WGCNA leverages similarities in gene expression patterns to construct co-expression networks and identify modules of genes that are closely associated with specific disease traits. As a result, it provides a solid foundation for subsequent candidate gene screening. In this study, WGCNA was performed on the expression matrix of IBD patient samples from the training set GSE3365 using the R package “WGCNA” (version 1.73).

First, hierarchical clustering was performed on the training set samples using the Euclidean distance of gene expression levels as the clustering criterion to detect outliers, thereby ensuring the reliability of subsequent analyses. Batch effects were corrected using the ComBat function in the sva package, which is a standard method for transcriptome data batch correction. Next, before constructing the co-expression network, it was necessary to select an optimal soft threshold (power) that makes the network more consistent with a scale-free topology and enhances its biological relevance. To this end, soft thresholds ranging from 1 to 20 were evaluated, and the relationship between each β value and both the scale-free topology fit index R² and the mean connectivity was analyzed. Based on the selected optimal soft threshold β = 7, a scale-free gene co-expression network was constructed, and genes with similar expression patterns were grouped into distinct modules, each distinguished by a different color. The minimum number of genes per module was set to 50, the pruning tree parameter to 4, and the module merging parameter to 0.15, which was set based on previous IBD-related WGCNA studies to ensure the independence of each module (Sun et al., 2024). Subsequently, the module eigengene (ME) and module membership (MM) were defined. Pearson correlation analysis was then performed to calculate the correlation coefficients and p-values between each module ME and IBD disease traits. Key modules with significant correlations were screened using the criteria of |r| ≥ 0.4 and P < 0.05. Finally, the “labeledHeatmap” function was used to display the correlations between modules and traits as a heatmap.

### 2.4 Candidate gene identification

To identify candidate genes associated with IBD that are also closely linked to the gut microbiota, we extracted three sets of genes. The first set consisted of 1,274 gut microbiota-associated genes from the gutMGene database (version 2.0; http://bio-computing.hrbmu.edu.cn/gutmgene). Specifically, all known human gut microbiota-derived small-molecule metabolites were first extracted from gutMGene v2.0 using the “Metabolite” browsing function, and their standardized SMILES molecular structures were retrieved from the PubChem database. Each metabolite SMILES was then submitted to the SwissTargetPrediction online platform (https://swisstargetprediction.ch/index.php), restricted to Homo sapiens, and only predicted target proteins with a probability score greater than 0.1 were retained to minimize false-positive predictions. The resulting target genes were compiled and deduplicated to obtain the final set of 1,274 gut microbiota-associated genes. The complete list of all 1,274 genes is provided as **Supplementary Table 1** for full transparency.

The second set comprised 885 DEGs from transcriptome analysis of IBD samples. The third set included 280 key module genes from WGCNA analysis. The gutMGene database is a comprehensive resource that integrates associations among gut microbiota, metabolites, and the host through manual literature curation and metabolic reconstruction techniques (Qi et al., 2025). Subsequently, an intersection analysis was performed using Venny software (version 2.1.0) to retain genes commonly present across all three datasets. The overlapping genes were visualized using the VennDiagram package (version 1.7.3), and a total of 19 core candidate genes were ultimately identified. These genes were designated as IBD gut microbiota-associated candidate genes.

### 2.5 Enrichment analysis

To investigate the functional roles of the 19 candidate genes, enrichment analyses of Gene Ontology (GO) and the Kyoto Encyclopedia of Genes and Genomes (KEGG) were performed using the R package clusterProfiler (version 4.12.6), restricted to Homo sapiens. The GO analysis covered biological processes (BP), cellular components (CC), and molecular functions (MF) to systematically characterize the functional annotations of the candidate genes. The Benjamini-Hochberg (BH) method was used to adjust p-values for both GO and KEGG enrichment analyses, and terms with an adjusted p-value < 0.05 were considered significantly enriched (Sun et al., 2024; Chaosheng et al., 2025). To enhance specificity, KEGG pathways were further filtered to retain only those containing at least three enriched genes (Zhu et al., 2023). The enrichment results were visualized using the ggplot2 package. For GO analysis, bar plots and circular plots were generated for the top five most significantly enriched terms in each category. For KEGG analysis, bubble plots and circular plots were generated for the top 19 most significant pathways.

### 2.6 Construction of protein-protein interaction network

To investigate the interactions among the proteins encoded by the candidate genes, a PPI network was constructed using the STRING database (https://cn.string-db.org/, Homo sapiens). A minimum combined confidence score of > 0.4 (a commonly used threshold in PPI network construction for IBD research) was applied to minimize false-positive interactions. Prior to visualization with Cytoscape software (version 3.10.3), all disconnected nodes were removed. In the resulting network, nodes represent proteins encoded by the candidate genes, edges denote PPIs, and node sizes are proportional to the degree of connectivity of each protein.

### 2.7 Machine learning for screening and validation of key genes

To identify core target genes with significant diagnostic value and regulatory importance for IBD from the 19 candidate genes, we employed three machine learning algorithms, namely LASSO regression, Boruta, and SVM-RFE, for cross-screening. Core target genes were then determined through Venn diagram intersection analysis. Finally, their reliability and specificity were validated via ROC curve analysis and expression validation.

#### 2.7.1 LASSO algorithm

During feature selection, we employed the least absolute shrinkage and selection operator (LASSO) regression method. This method introduces an L1 penalty term, defined as the sum of the absolute values of the regression coefficients, into the ordinary least squares loss function. The regularization parameter λ governs the penalty strength: a larger λ exerts stronger shrinkage, progressively driving the coefficients of less important features toward zero. This property enables LASSO to perform simultaneous model fitting and feature selection, effectively mitigating overfitting in high-dimensional data and making it particularly suitable for biomarker identification. The optimal λ value was determined via ten-fold cross-validation using the ‘glmnet’ package in R (version 4.1.3), and the penalty factor was set to 1, yielding an optimal λ.min of 0.02628. Features with non-zero coefficients at this λ value were retained as core features, ultimately identifying five representative genes from the candidate gene pool.

#### 2.7.2 Boruta machine learning analysis

Feature importance was assessed using the Boruta algorithm as implemented in the Boruta package (version 9.0.0). This method generates shadow features, which are random permutation copies of the original features, and compares the importance of the original features with that of the shadow features using a random forest based importance measure, with the number of random forest trees set to 1000 and the significance level set to 0.05. Features with importance scores significantly higher than the maximum shadow feature score were classified as “confirmed” (important), whereas those with significantly lower scores were classified as “rejected” (unimportant). This approach achieves robust feature selection by considering both relevance and statistical significance. Among the 19 candidate genes, 16 were confirmed as important features and retained for subsequent analysis.

#### 2.7.3 SVM-RFE algorithm analysis

SVM-RFE is a sequential backward selection algorithm based on the maximum margin principle of support vector machines. It iteratively trains the model on the sample data, ranks the features by importance scores, removes the feature with the smallest score, and retrains the model using the remaining features. This process continues until the desired number of features is selected. SVM-RFE analysis was performed on the 19 candidate genes using the R package “caret” (version 7.0-1), with the radial basis function (RBF) used as the kernel function. The ‘caret’ package was used to perform 10-fold cross-validation to determine the optimal number of feature genes, ultimately identifying four feature genes.

#### 2.7.4 Identification and validation of core target genes

By taking the intersection of the five genes identified by LASSO regression, the 16 genes identified by the Boruta algorithm, and the four genes identified by the SVM-RFE algorithm, we ultimately identified three core hub genes, namely C5AR1, KDM2A, and HCAR3, as candidate key genes for IBD.

To evaluate the diagnostic value of these candidate key genes, receiver operating characteristic (ROC) curve analysis was performed using the R package “pROC” (version 1.19.0.1) in both the training set and the validation set, and the area under the curve (AUC) was calculated. Genes with an AUC greater than 0.7 were selected for subsequent analysis. Furthermore, we have now reported the detailed ROC performance metrics including optimal cut-off values, sensitivity, and specificity for each hub gene in both the training and validation sets, which have been added as **Supplementary Table 2** in the revised manuscript. Differences in the expression levels of the candidate key genes between the IBD patient group and the control group were analyzed, with statistical significance set at P < 0.05.

### 2.8 Molecular docking verification

To evaluate the binding potential between the proteins encoded by the identified hub genes and key gut microbiota-derived metabolites, we performed molecular docking analysis. The three-dimensional crystal structures of C5AR1 (PDB ID: 5O9H) and HCAR3 (PDB ID: 8JEI) were obtained from the Protein Data Bank (PDB; https://www.rcsb.org/). The metabolite structures were retrieved from the PubChem database (https://pubchem.ncbi.nlm.nih.gov/), and the structures of 1,3-Diphenylpropan-2-Ol (PubChem CID: 3061846) and 3-Indolepropionic Acid (PubChem CID: 3744) were retrieved. All protein structures were preprocessed using PyMOL (version 3.1.6.1) to remove water molecules and ligands, and AutoDock Tools (version 1.5.6) was used to add polar hydrogen atoms and assign Gasteiger charges. Metabolite structures were preprocessed using ChemDraw 2020 to add hydrogen atoms, and their conformations were optimized using the MMFF94 force field.

Molecular docking simulations were performed using the CB-Dock2 platform (https://cadd.labshare.cn/cb-dock2/), which employs the CurPocket algorithm to automatically identify multiple potential binding pockets and generate corresponding grid box parameters for blind docking. To account for the stochastic variation inherent in the AutoDock Vina algorithm, we performed 10 independent docking runs for each ligand-target combination, and the exhaustiveness parameter was set to 8 (the platform default) to ensure thorough conformational sampling. The grid boxes for each protein–metabolite pair were defined as follows: for C5AR1 with 1,3-Diphenylpropan-2-Ol, the grid box center was set at (51, 45, 43) with dimensions of 19 Å × 19 Å × 19 Å; for HCAR3 with 3-Indolepropionic Acid, the grid box center was set at (140, 121, 90) with dimensions of 19 Å × 19 Å × 19 Å.

Binding energies below 0 kcal·mol^-^¹ indicate spontaneous binding between the receptor and ligand, whereas values below −5 kcal·mol^-^¹ reflect strong and stable interactions. In general, a lower binding energy corresponds to a higher binding affinity. All docking results are reported as mean ± standard deviation (SD) from 10 independent runs to assess reproducibility.

### 2.9 Drugability and toxicity prediction of metabolites

The pharmacological properties of the metabolites were evaluated using the SwissADME platform (https://www.swissadme.ch/), which predicts absorption, distribution, metabolism, and excretion (ADME) properties along with drug likeness. The assessment focused on compliance with Lipinski’s rule of five, oral bioavailability, and other key drug development indicators. A high throughput evaluation of toxicological properties was performed using the ADMETlab platform (version 2.0; https://admetmesh.scbdd.com/). This provided a comprehensive assessment of clinical translation potential for the core metabolites, covering hERG channel blockade risk, hepatotoxicity (H-HT), drug induced liver injury (DILI), carcinogenicity, and skin sensitization.

## 3 Results

### 3.1 Identification and correlation analysis of IBD-related differential genes

**Figure 1:**
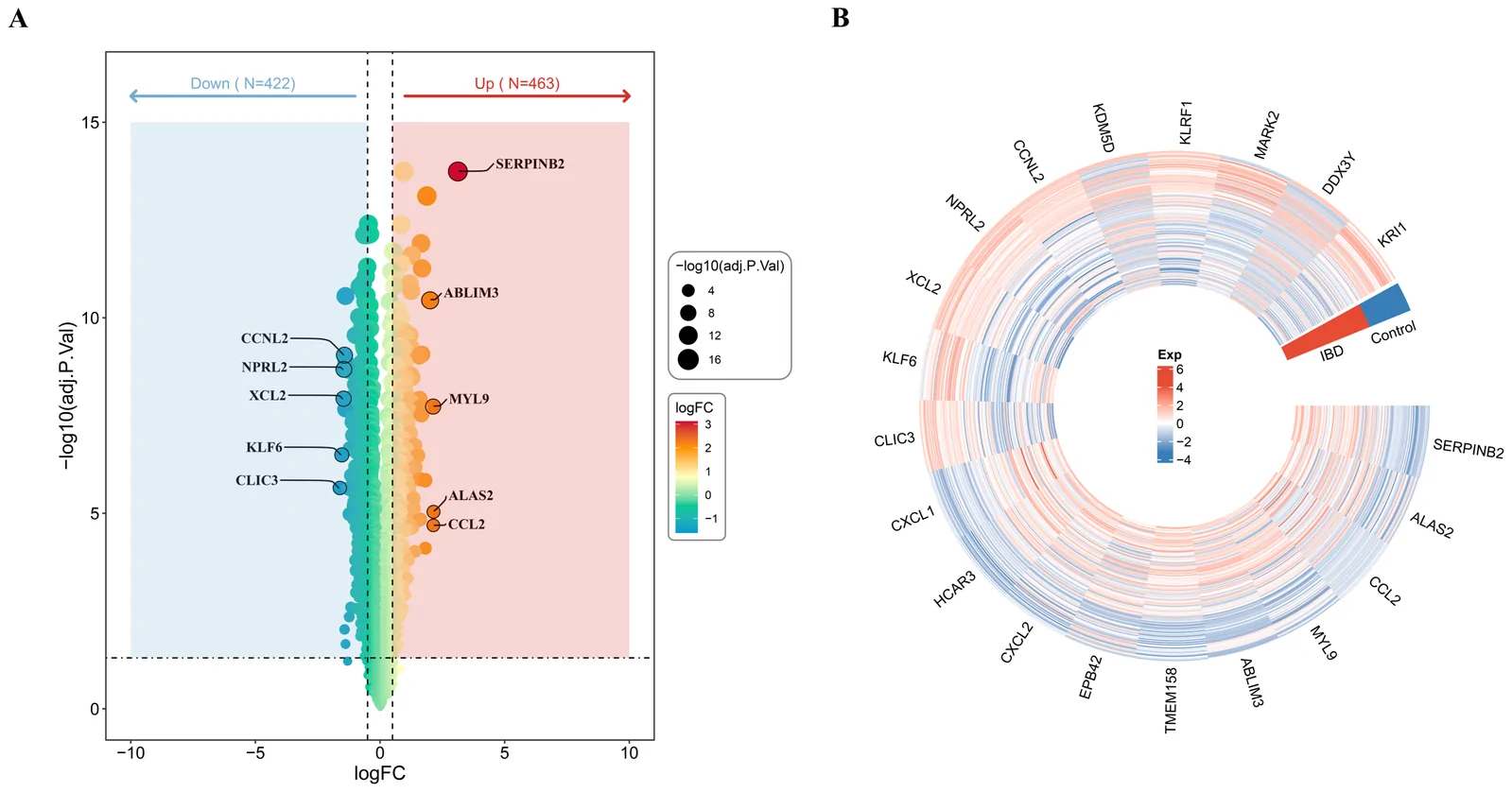
Screening of core DEGs in IBD. **(A)** Volcano plot of DEGs between the IBD group and healthy controls from the GEO database. The horizontal dashed line indicates the threshold of |log_2_(fold change)| = 0.5, and the vertical dashed line represents −log_10_(0.05) = 1.3. Red, blue, and gray dots denote upregulated, downregulated, and non-DEGs, respectively. The top five upregulated and top five downregulated genes ranked by fold change are labeled. **(B)** Heatmap illustrating the expression profiles of DEGs. Red represents high expression, while blue represents low expression. All DEGs remained statistically significant after Benjamini-Hochberg correction (adjusted p < 0.05). DEGs, differentially expressed genes; IBD, inflammatory bowel disease; GEO, Gene Expression Omnibus.

According to the screening criteria of p < 0.05 and |log_2_FC| > 0.5, a total of 885 DEGs were identified between the IBD group and the control group. All genes remained statistically significant after Benjamini-Hochberg correction (adjusted p < 0.05). Among them, 463 genes were upregulated and 422 genes were downregulated in the IBD group. Of these DEGs, the top 5 upregulated genes (e.g., SERPINB2, ABLIM3) were mainly involved in inflammatory response, and the top 5 downregulated genes (e.g., CCNL2, NPRL2) were associated with intestinal barrier function, which is consistent with the pathological characteristics of IBD. To visualize the expression patterns, a volcano plot (**Figure 1A**) and a heatmap (**Figure 1B**) were generated. In the volcano plot, upregulated genes are shown in red and downregulated genes in blue. In the heatmap, red indicates high expression and blue indicates low expression. Both plots clearly revealed expression differences between the two groups and demonstrated good inter-sample clustering. Collectively, these 885 DEGs reflect the key molecular changes characteristic of IBD and provide a foundational basis for subsequent screening of gut microbiota-associated candidate genes.

### 3.2 Screening of key module genes by WGCNA analysis

#### 3.2.1 Sample hierarchical clustering and outlier detection

**Figure 2:**
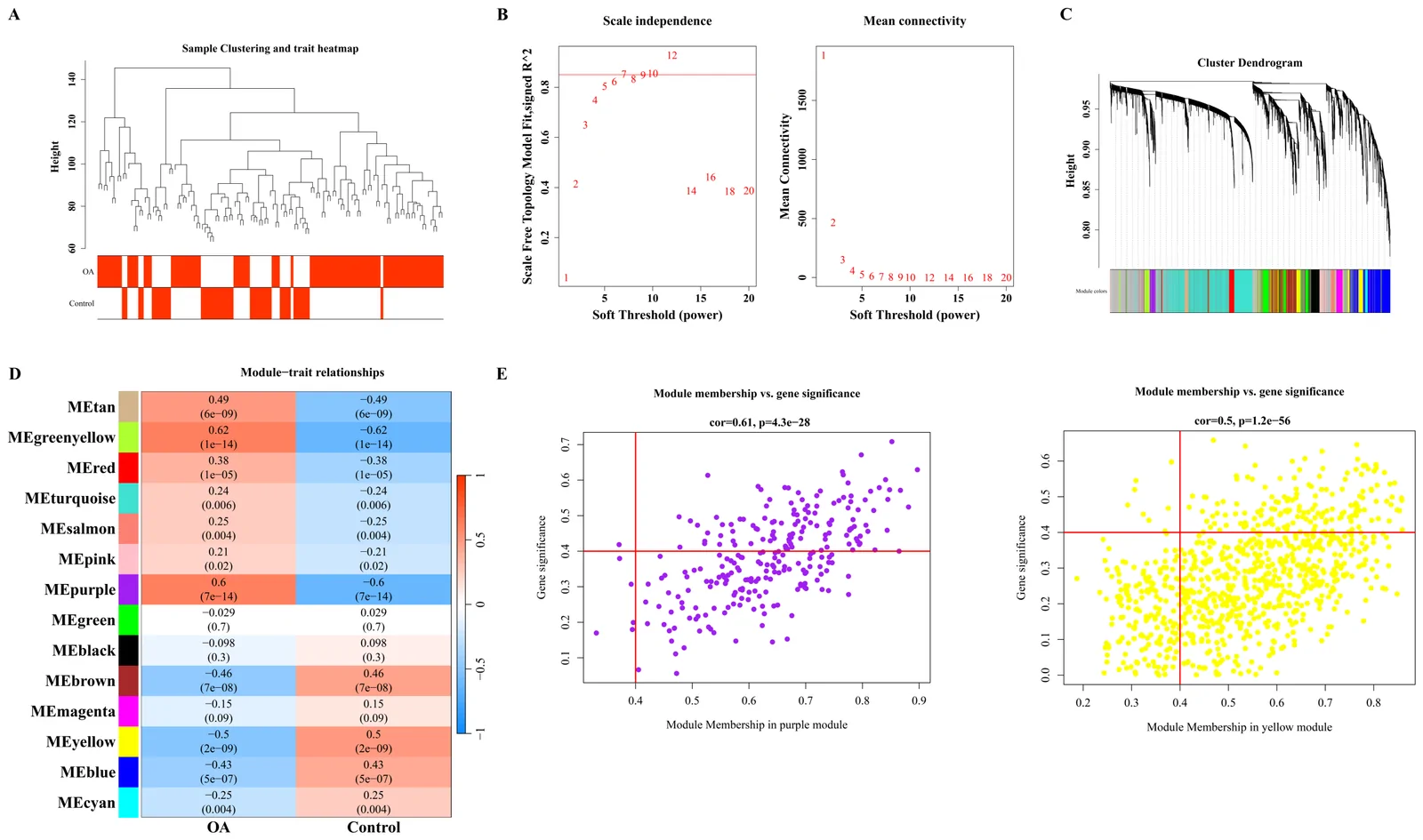
WGCNA analysis identifies key module genes. **(A)** Sample-level clustering. In the clustering tree, each branch represents an individual sample, and the vertical axis indicates the Euclidean distance of sample expression levels, where a larger distance reflects greater dissimilarity in expression patterns between samples. No significant outliers were detected, confirming the suitability of the dataset for subsequent WGCNA analysis. **(B)** Selection of the optimal soft threshold. The horizontal axis represents the power parameter value. In the left plot, the vertical axis shows the scale-free fit index (signed R²), which assesses the scale-free topology of the network. In the right plot, the vertical axis represents the mean connectivity, defined as the average adjacency function of all genes within a given module. Combined analysis of the two plots indicates that β = 7 is the optimal soft threshold, satisfying the requirements of a scale-free network. **(C)** Co-expression module identification. The upper panel shows a hierarchical clustering dendrogram of genes, and the lower panel displays the corresponding gene modules, with distinct colors representing different modules. Genes clustered on the same branch are assigned to the same module, clearly illustrating the modular classification. **(D)** Module and feature correlation heat map. The color blocks on the far left represent individual modules, and the color bar on the far right indicates the correlation range. The color intensity in the central section reflects the strength of the correlation, with red denoting positive correlation and blue denoting negative correlation. The numbers in each cell represent the correlation coefficient and significance level, providing an intuitive visualization of the association between each module and IBD traits. **(E)** MM-GS correlation scatter plot. The purple module (left) exhibited the highest positive correlation with IBD disease traits (R = 0.6), whereas the yellow module (right) showed the strongest negative correlation (R = −0.5). Genes within these two modules were selected as module genes for further analysis. The correlations between MM and GS were assessed to evaluate the relationships of module genes with their respective modules and with disease traits. MM, module membership; GS, gene significance.

In the clustering tree, each branch represents an individual sample, and the vertical axis denotes the Euclidean distance of expression levels between samples, where a greater distance reflects larger differences in expression patterns. As shown in **Figure 2A**, no significant abnormalities or obvious outliers were observed among the sample branches. All samples were therefore retained for subsequent analyses.

#### 3.2.2 Selection of the optimal soft threshold

In this study, candidate soft thresholds ranging from 1 to 20 were evaluated, and the relationship between the soft threshold β and both the scale-free topology fit index R² and the mean connectivity was analyzed (**Figure 2B**). In both plots, the horizontal axis represents the power parameter value. In the left plot, the vertical axis represents the scale-free fit index (signed R²), which assesses the scale-free property of the network. In the right plot, the vertical axis represents the mean connectivity, defined as the average adjacency function of all genes within a given module.

While the general field standard recommends SFT.R.sq ≥ 0.8, we adopted a more stringent threshold of 0.85 to ensure greater topological stability and robustness of the co-expression network. At β = 6, the SFT.R.sq value was only 0.823, which did not meet our preset criterion. When β was increased to 8, the fit coefficient decreased to 0.833, indicating that excessively high thresholds may eliminate too many gene-gene associations and compromise the overall network structure. At β = 7, the scale-free topology fit index reached SFT.R.sq = 0.852, with a mean connectivity of mean.k = 6.93, a median connectivity of median.k = 2.71, and a maximum connectivity of max.k = 89.6. These values indicate that the network at β = 7 best conforms to a scale-free distribution while maintaining moderate mean connectivity, thereby preserving the integrity of gene association information without excessive pruning. Therefore, β = 7 was determined as the optimal soft threshold for this dataset.

#### 3.2.3 Identification of co-expression modules

As shown in **Figure 2C**, WGCNA identified a total of nine co-expression modules, excluding the grey module which contains unassigned genes. In the figure, the upper part displays the hierarchical clustering dendrogram of genes, while the lower part presents the gene modules, with distinct colors representing different modules. Genes with closer distances that are clustered into the same branch are assigned to the same module, clearly illustrating the modular divisions. The specific expression patterns observed in each module lay a solid foundation for subsequent module-trait association analyses.

#### 3.2.4 Key module screening and module gene identification

Pearson correlation analysis was performed to calculate the correlation coefficients and p-values between MEs and IBD disease traits. Key modules with significant correlations were screened using the criteria of |r| ≥ 0.4 and P < 0.05. The “labeledHeatmap” function was used to visualize the correlations between modules and traits as a heatmap (**Figure 2D**). In this heatmap, the color blocks on the far left represent individual modules, while the color bar on the far right indicates the correlation range. The intensity of the color in the middle section reflects the strength of the correlation, with red representing positive correlation and blue representing negative correlation. The numbers in each cell denote the correlation coefficient and significance, providing an intuitive presentation of the association between each module and IBD traits.

The analysis results showed that the purple module exhibited the highest positive correlation with IBD disease traits (R = 0.6). The purple module genes were mainly enriched in TNF/NF-κB signaling pathways (subsequently verified by GO/KEGG analysis), which are core pathways of IBD inflammation, indicating that these module genes are closely related to IBD pathogenesis. In contrast, the yellow module showed the highest negative correlation (R = −0.5). Genes within these two modules were therefore selected as module genes. Further analysis was conducted on MM and gene significance (GS) for disease traits, with thresholds set at |cor.geneModuleMembership| > 0.6 and |cor.geneTraitSignificance| > 0.6. Ultimately, 280 key module genes were obtained, including 117 from the purple module and 163 from the yellow module, for subsequent candidate gene screening (**Figure 2E**).

### 3.3 Candidate gene identification

**Figure 3:**
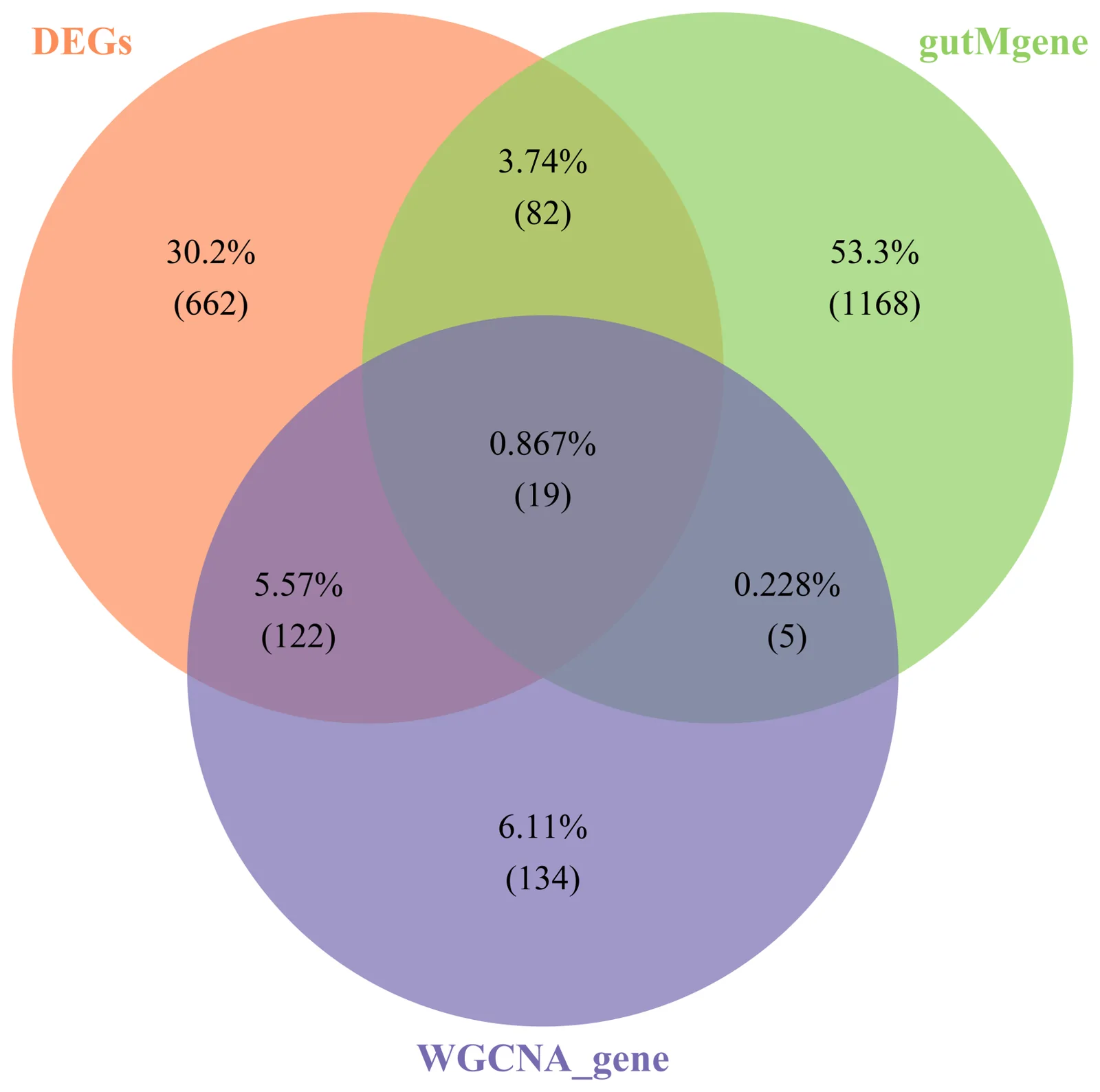
Venn diagram analysis of overlapping DEGs, gut microbiota-related genes and WGCNA key module genes. Core candidate genes were defined as the intersection of three distinct gene sets: IBD-related DEGs, gut microbiota-associated genes, and WGCNA key module genes. Only genes shared across all three sets were retained for subsequent analysis. DEGs, differentially expressed genes; IBD, inflammatory bowel disease.

From the differential expression analysis, we identified 885 DEGs between the IBD patients and healthy controls. Separately, we obtained 1,274 gut microbiota-associated genes from the gutMGene database and screened 280 key module genes through WGCNA. To pinpoint candidate genes potentially mediating the interaction between gut microbiota and IBD pathogenesis, we performed an intersection analysis on these three gene sets. This analysis subsequently yielded 19 core candidate genes (**Figure 3**). Designated as IBD gut microbiota-associated candidate genes, these genes serve as a key bridge linking gut microbiota dysbiosis to IBD pathogenesis and establish a foundation for subsequent functional enrichment and hub gene screening.

### 3.4 Functional enrichment analysis of candidate genes

**Figure 4:**
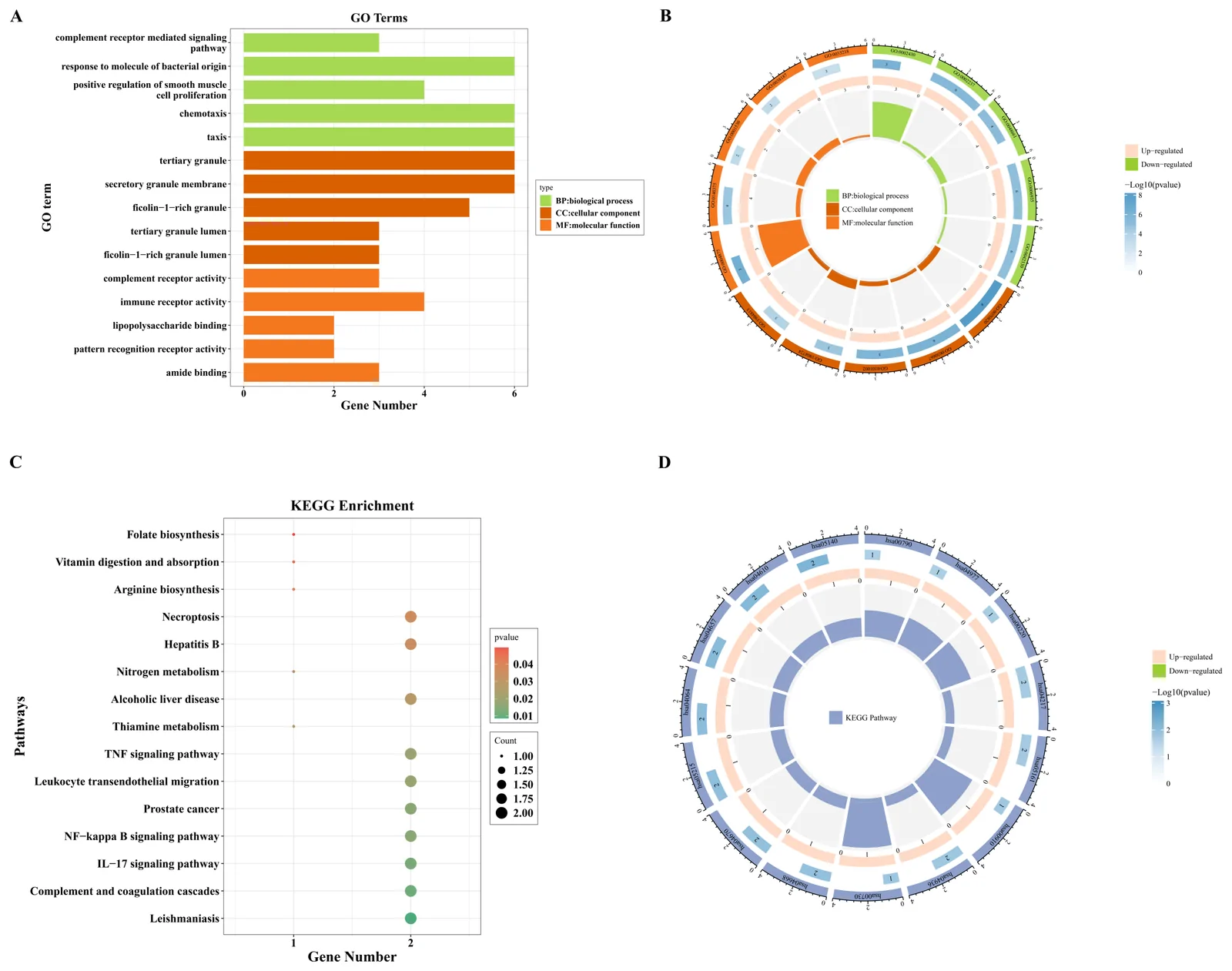
Functional enrichment analysis of candidate genes. **(A)** GO bar plot of candidate genes. The x-axis shows the number of genes mapped to each term, and the y-axis lists the term names. **(B)** GO circle plot. The concentric layers from outer to inner are organized as follows. The first layer displays GO functional IDs, categorized into three main ontologies: BP, CC, and MF. The second layer uses color intensity to indicate significance levels, with the segment size and numerical values reflecting the number of genes enriched in each term. The third layer shows the counts of upregulated and downregulated genes within each term, distinguished by different colors. The innermost layer consists of color blocks representing distinct functions, where the size of each block indicates the Rich Factor, with larger values denoting greater enrichment. **(C)** KEGG bubble plot of candidate genes. The x-axis represents the number of genes mapped to each pathway, and the y-axis lists the pathway names. Bubble size reflects the number of enriched genes, while color intensity indicates −log_10_(p-value), with darker colors representing higher significance. **(D)** KEGG circle plot. The concentric layers from outer to inner are organized as follows. The first layer displays KEGG pathway IDs. The second layer uses color intensity to indicate significance levels, with the segment size and numerical values reflecting the number of genes enriched in each pathway. The third layer shows the counts of upregulated and downregulated genes within each pathway, distinguished by different colors. The innermost layer consists of color blocks representing distinct pathways, where the size of each block indicates the Rich Factor. The Benjamini-Hochberg method was used for p-value adjustment, and an adjusted p < 0.05 was set as the significance threshold. GO, Gene Ontology; BP, biological process; CC, cellular component; MF, molecular function; KEGG, Kyoto encyclopedia of genes and genomes.

To investigate the functional roles of the candidate genes, GO and KEGG enrichment analyses were performed using the clusterProfiler package. The GO analysis identified 533 significantly enriched terms, including 450 BP, 23 CC, and 60 MF. The top five most significant terms in each category were visualized using bar plots and pie charts (**Figures 4A, B**). In the bar plot, the x-axis shows the number of genes mapped to each term, and the y-axis lists the term names. The pie chart consists of four concentric layers, displaying the GO term ID, significance level, gene count (including both upregulated and downregulated genes), and enrichment factor value. The Benjamini-Hochberg method was used to adjust p-values. As shown in **Figures 4A, B**, the candidate genes were significantly enriched in BP terms such as response to molecule of bacterial origin, chemotaxis, and taxis (adjusted p < 0.001), with the top 5 enriched terms mainly related to microbial response and inflammatory regulation. At the CC level, enrichment was primarily observed in the tertiary granule and secretory granule membrane, which are closely associated with immune cell function. At the MF level, enriched terms included complement receptor activity, immune receptor activity, lipopolysaccharide binding, pattern recognition receptor activity, and amide binding, all of which are involved in the regulation of IBD-related inflammatory signaling pathways.

KEGG analysis identified 19 significantly enriched pathways (adjusted p < 0.05), of which the top 15 ranked by p-value were selected for visualization. As shown in **Figures 4C, D**, the candidate genes were significantly enriched in pathways including necroptosis, hepatitis B, the TNF signaling pathway, the NF-κB signaling pathway, and the IL-17 signaling pathway. Among these, the TNF, NF-κB, and IL-17 signaling pathways are core regulators of IBD inflammatory responses, providing key directions for further elucidation of the “gut microbiota-metabolite-gene” regulatory mechanism and the screening of intervention targets. In the bubble plot (**Figure 4C**), the x-axis represents the number of genes mapped to each pathway, and the y-axis lists the pathway names. Additionally, a circular plot (**Figure 4D**) was generated, with its structural interpretation following the same principles as those used for the GO analysis.

To assess whether the stringent triple-intersection strategy resulted in excessive loss of biologically relevant genes, we performed a sensitivity analysis by conducting independent GO enrichment analyses on the WGCNA-derived module genes alone (n = 280). As shown in **Supplementary Figure 2**, the WGCNA-only recapitulated the same core biological processes as those identified using our 19 candidate genes, including response to bacterial-derived molecules, tertiary granule and secretory granule membrane components, lipopolysaccharide binding, and immune receptor activity. This concordance confirms that our stringent filtering did not distort the key functional landscape, and that the 19 candidate genes represent a high-confidence core set enriched for gut microbiota-IBD interactions.

### 3.5 PPI network construction

**Figure 5:**
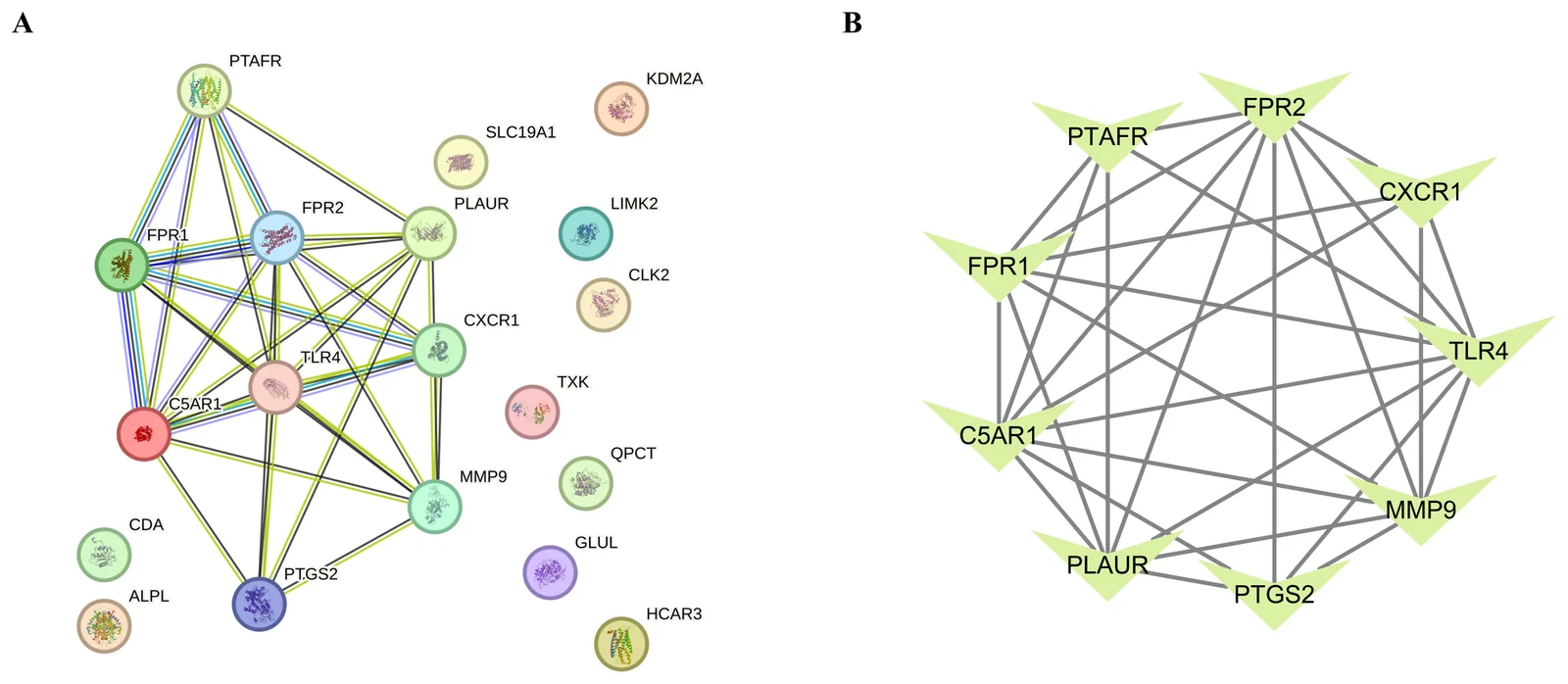
PPI network analysis of candidate genes. **(A)** PPI network constructed using the STRING database. **(B)** Optimized and visualized network using Cytoscape. In both panels, nodes represent proteins encoded by candidate genes, edges denote protein-protein interactions, and node size is proportional to the degree of connectivity. PPI, protein-protein interaction.

To analyze the interactions among proteins encoded by the 19 candidate genes and to identify core nodes within the regulatory network, we submitted the candidate genes to the STRING database, setting an interaction confidence threshold greater than 0.4. To this end, high-confidence PPIs were screened and subsequently visualized using Cytoscape software. This process resulted in a PPI network comprising 9 core protein nodes and 30 interaction edges (**Figures 5A, B**). This network effectively highlights key protein nodes implicated in IBD pathogenesis, thereby laying a solid foundation for subsequent machine learning-based hub gene screening.

### 3.6 Machine learning for screening and validation of key genes

#### 3.6.1 Screening of key genes by three machine learning algorithms

**Figure 6:**
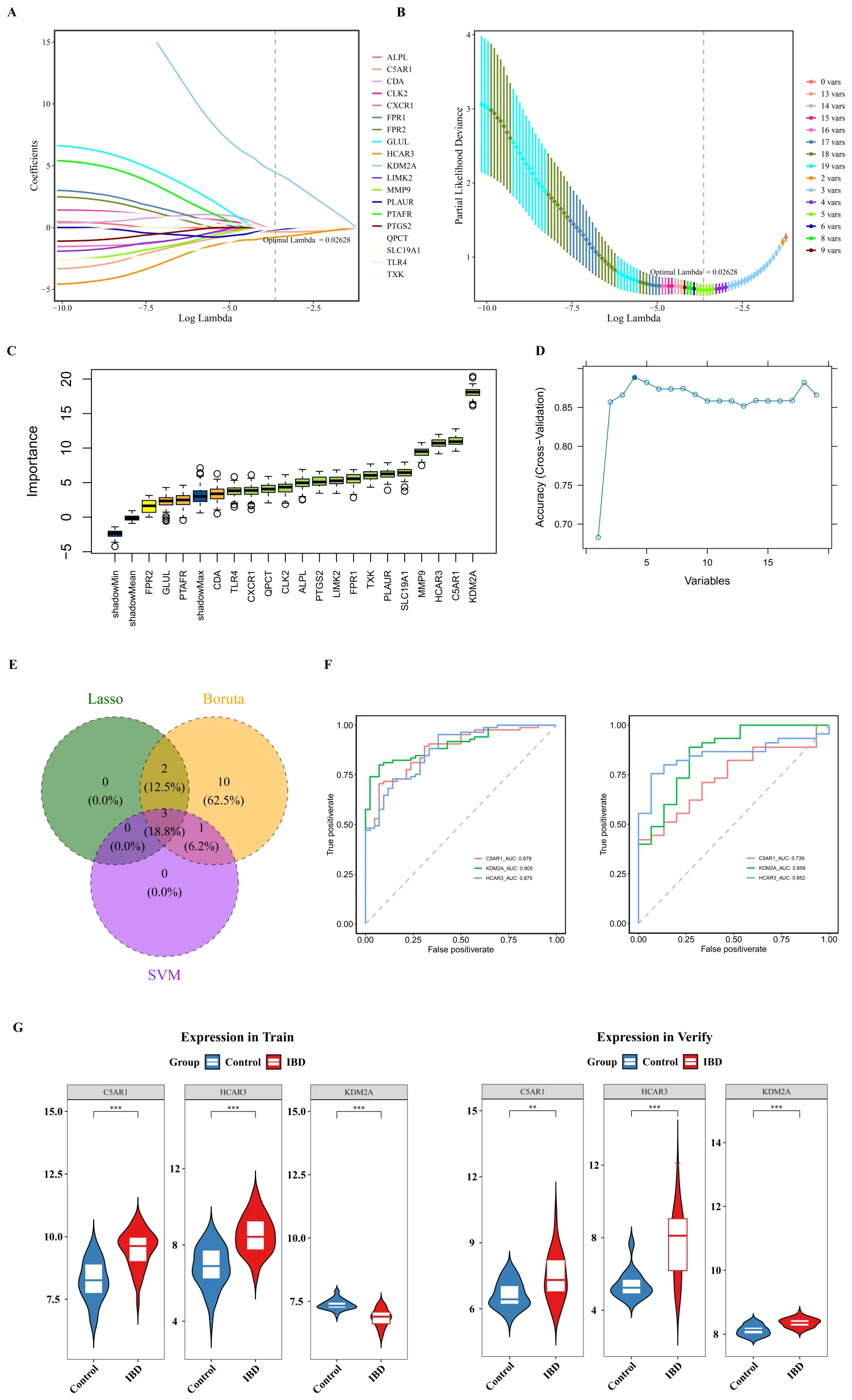
Identification of core key genes using three machine learning algorithms. **(A)** Coefficient path diagram of the LASSO regression model. The x-axis represents the logarithm of the regularization parameter λ (log λ), and the y-axis shows the regression coefficients of variables. As λ gradually increases, the coefficients shrink toward zero, and variables with zero coefficients are eliminated when the optimal λ is reached. **(B)** LASSO cross-validation curve. The x-axis represents log λ, and the y-axis represents the model error. The left vertical dashed line indicates the optimal λ value (lambda.min = 0.02628) corresponding to the minimum model error. Under this optimal parameter, five feature genes were identified and retained: C5AR1, KDM2A, HCAR3, PLAUR, and SLC19A1. **(C)** Gene importance ranking plot generated by the Boruta algorithm. The x-axis represents individual genes, and the y-axis shows feature importance scores. Blue bars denote shadow reference features, yellow bars indicate tentative features, and green bars represent confirmed important features. A total of 16 genes were identified as important by this algorithm. **(D)** SVM-RFE algorithm analysis. The x-axis represents the number of feature genes, and the y-axis indicates the model’s prediction accuracy. As the number of feature genes increases, prediction accuracy initially rises and then stabilizes, peaking at four feature genes. **(E)** Among the five genes identified by LASSO, the 16 genes identified by Boruta, and the four genes identified by SVM-RFE, three overlapping genes—namely C5AR1, KDM2A, and HCAR3—were identified as core hub genes jointly validated by all three algorithms. **(F)** ROC curves and AUC values of the core target genes in the training set (left) and validation set (right). All three genes achieved AUC values greater than 0.7 in both datasets, indicating excellent diagnostic performance for IBD. **(G)** Expression levels of the candidate key genes in the training set (left) and validation set (right). In the violin plots, statistical significance is denoted as follows: ****p < 0.0001; ***p < 0.001; **p < 0.01; *p < 0.05; ns, p > 0.05.

The performance of the LASSO regression model and its feature selection outcome depend on the regularization parameter λ. To determine the optimal λ value, we generated a coefficient path plot and a CV deviance plot. In **Figure 6A**, the x-axis represents the logarithm of λ, and the y-axis shows the variable coefficients (i.e., regression coefficients). As λ increases, the coefficients gradually shrink toward zero. At the optimal λ value, variables with coefficients of zero are excluded from the model. In **Figure 6B**, the x-axis also represents the logarithm of λ, while the y-axis represents the model error. The optimal λ is indicated by the vertical dashed line, which marks the point of minimum model error. The optimal regularization parameter was determined using ten-fold cross-validation, yielding a λ.min value of 0.02628. After removing redundant genes with coefficients near zero, five feature genes were ultimately retained: C5AR1, KDM2A, HCAR3, PLAUR, and SLC19A1.

**Figure 6C** presents the gene importance ranking plot generated by the Boruta algorithm. The horizontal axis represents individual genes, and the vertical axis represents their corresponding importance scores. In the plot, blue denotes shadow reference features, yellow denotes tentative features, and green denotes confirmed important features. Genes are arranged from left to right in ascending order of importance. Using this algorithm, 16 significant genes were identified: C5AR1, KDM2A, HCAR3, PLAUR, FPR1, MMP9, LIMK2, PTGS2, TXK, TLR4, ALPL, CLK2, SLC19A1, QPCT, CDA, and CXCR1.

To further refine the candidate gene set, we performed SVM-RFE analysis on the 19 candidate genes. As shown in **Figure 6D**, the highest model prediction accuracy was achieved when the number of feature genes was set to four. These four feature genes were identified as KDM2A, C5AR1, HCAR3, and MMP9.

Subsequently, a cross-analysis was performed among the five feature genes identified by LASSO regression, the 16 key genes identified by the Boruta algorithm, and the four feature genes identified by the SVM-RFE algorithm. This cross-analysis yielded three core hub genes: C5AR1, KDM2A, and HCAR3. The overlapping relationships among the three gene sets are illustrated in the Venn diagram (**Figure 6E**). These three genes, validated as core targets by all three algorithms, represent key molecules for regulating and intervening in IBD-associated gut microbiota.

#### 3.6.2 Identification and validation of core target genes

ROC curve analysis was performed using the ‘pROC’ package, and the significance of AUC values was tested using the DeLong test (p < 0.001 for all three hub genes). All three core genes exhibited consistent expression trends and high AUC values (>0.7) in both the training and validation sets, indicating high stability and potential clinical diagnostic value, thereby effectively overcoming the limitations of single-dataset analysis (**Figure 6F**). To provide clinically interpretable diagnostic metrics, we further calculated the optimal cut-off values, sensitivity, and specificity for each hub gene in both cohorts, and these detailed performance metrics are summarized in **Supplementary Table 2**.

To benchmark our hub genes against established IBD biomarkers, we evaluated TNF and IL6 in both datasets (IL23R was excluded due to its lack of concurrent presence across the two datasets). ROC analysis showed that TNF and IL6 had AUC values below 0.7 in the training set but above 0.7 in the validation set, indicating inconsistent performance across cohorts (**Supplementary Figure 3**). In contrast, our hub genes, particularly C5AR1 and HCAR3, maintained robust AUC values above 0.7 in both datasets, suggesting superior and more stable diagnostic utility compared to these conventional markers.

Further analysis of expression level differences between the IBD patient group and the control group (P < 0.05) revealed that, in the training set, KDM2A was significantly downregulated in the disease group, whereas C5AR1 and HCAR3 were significantly upregulated (**Figure 6G**). In the validation set, the expression trends of C5AR1 and HCAR3 were consistent with those observed in the training set and remained statistically significant, indicating that these two feature genes exhibit stable expression patterns between disease and control groups across different datasets. Therefore, C5AR1 and HCAR3 were designated as key genes.

### 3.7 The construction of the “gut microbiota-metabolite-target” interaction network

**Figure 7:**
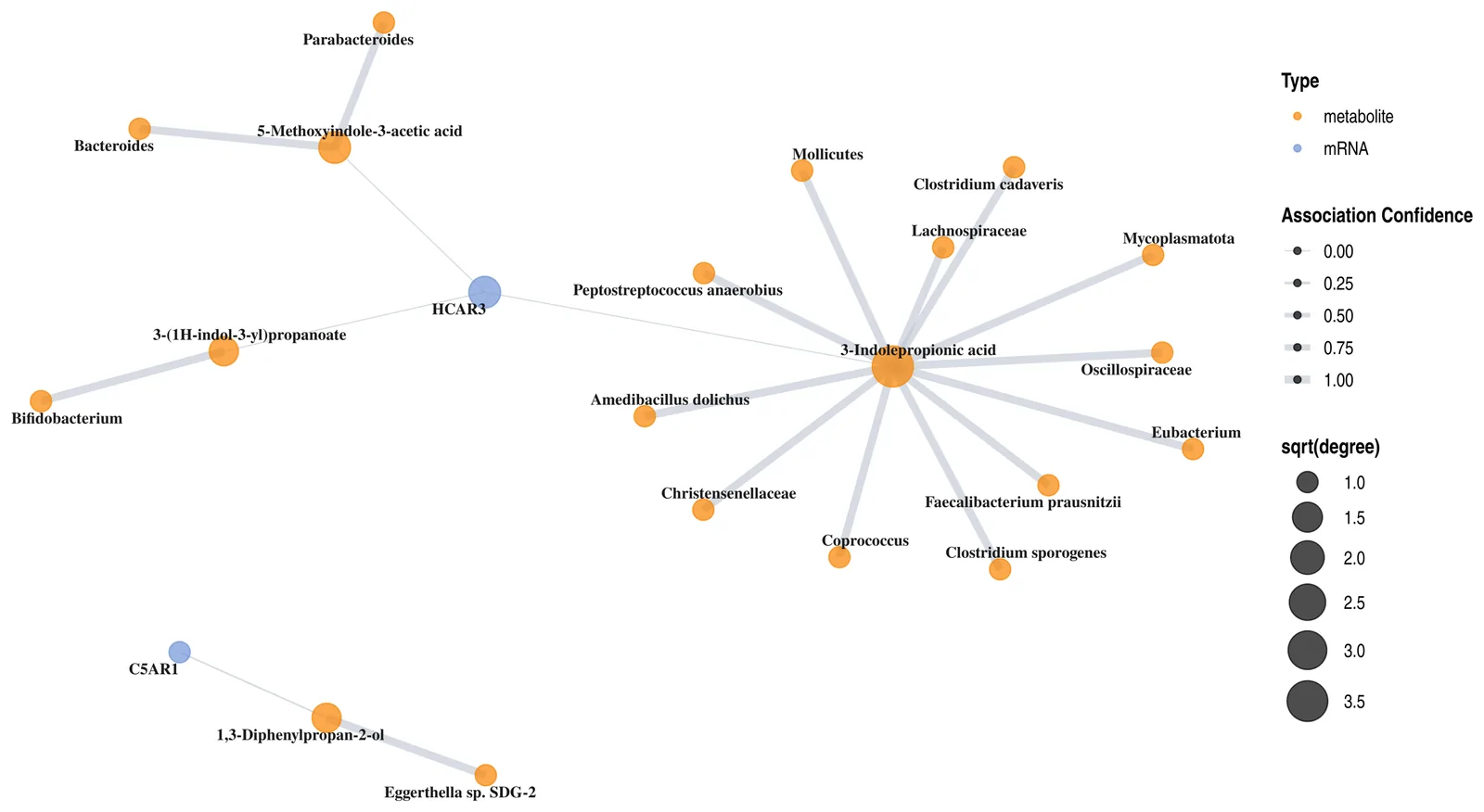
The microbiota-metabolite-target network. In the network, blue nodes represent target genes, orange nodes represent metabolites, and green nodes represent gut microbiota. Edges between nodes denote the interactions or regulatory relationships among these components.

This network diagram illustrates the “gut microbiota-metabolite-target” regulatory axis (**Figure 7**). The network shows that the two core target genes correspond to 55 metabolites, which in turn are associated with 75 gut microbial members. In the diagram, blue nodes represent the core target genes, orange nodes represent microbial metabolites, green nodes represent gut microbial communities, and edges denote direct regulatory interactions among these components. Collectively, this network visually presents the regulatory relationships connecting the gut microenvironment, metabolites, and targets, clearly revealing how gut microbiota may interact with core target genes through the production of specific metabolites to participate in IBD progression.

### 3.8 The evaluation of molecular docking

**Figure 8:**
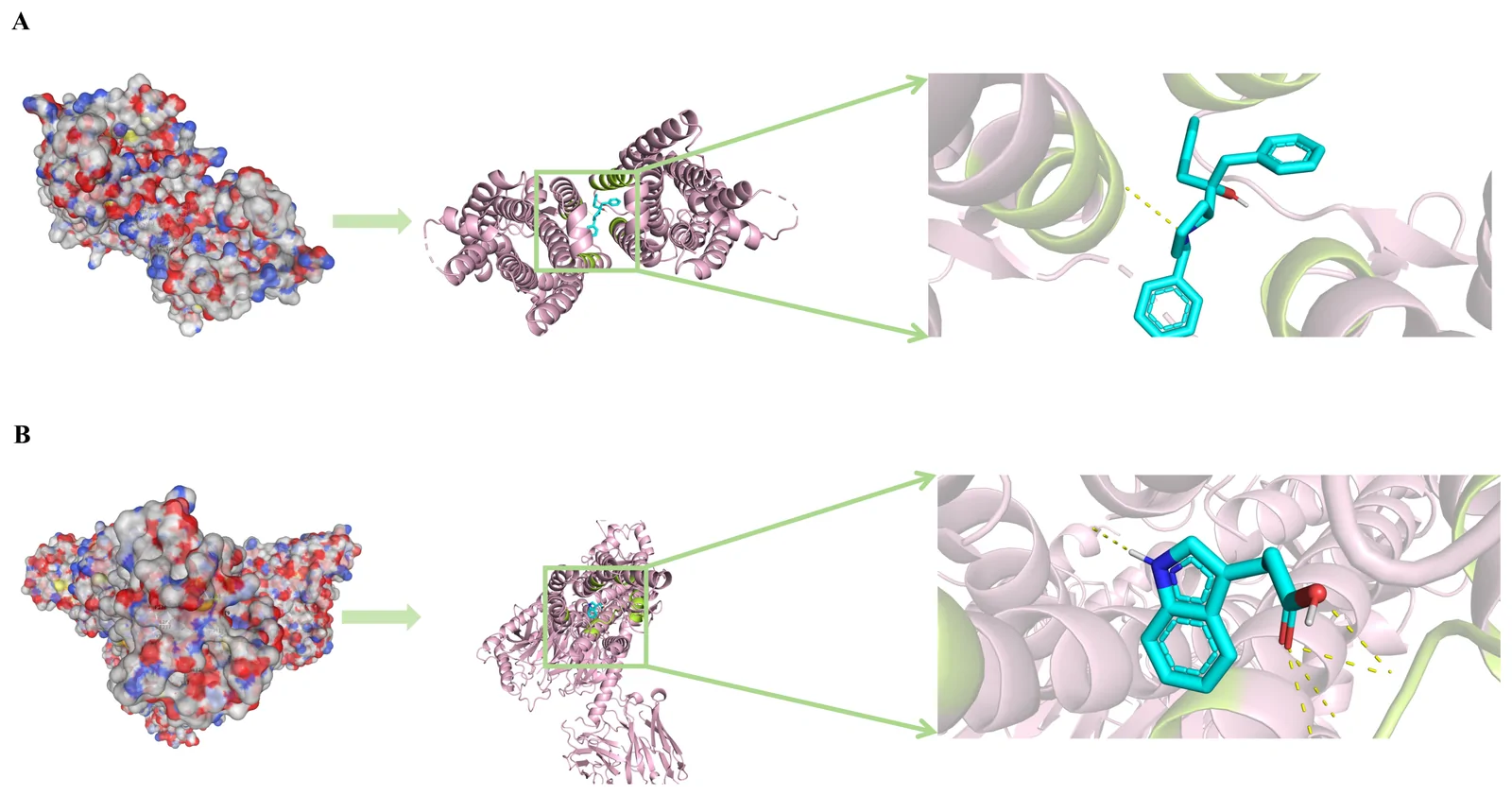
Molecular docking analysis of hub proteins with representative metabolites. **(A)** Binding conformation of C5AR1 with 1,3-Diphenylpropan-2-Ol, showing a binding energy of -7.87 ± 0.83 kcal/mol. **(B)** Binding conformation of HCAR3 with 3-Indolepropionic Acid, showing a binding energy of -6.35 ± 0.70 kcal/mol. For each panel, from left to right: the first image shows the electrostatic surface potential of the target protein (red: negatively charged; blue: positively charged; gray: neutral); the middle image displays the three-dimensional conformation of the target protein in blue; the right image provides a magnified view of the ligand binding site, where the metabolite is shown in light blue, hydrogen bonds are indicated by yellow dashed lines, and the light green area marks the binding interface. Both docking pairs exhibited binding energies below −5.0 kcal/mol, indicating stable and spontaneous interactions. All docking conformations were visualized using PyMOL.

As shown in **Table 1** and **Figure 8**, the binding energy between C5AR1 and 1,3-Diphenylpropan-2-Ol was -7.87 ± 0.83 kcal/mol (mean ± SD from 10 independent runs), and that between HCAR3 and 3-Indolepropionic Acid was -6.35 ± 0.70 kcal/mol. Both mean values were below -5.0 kcal/mol, indicating stable and spontaneous binding between the core metabolites and their respective target proteins. The small standard deviations demonstrate good reproducibility across independent docking runs. Among these, 1,3-Diphenylpropan-2-Ol exhibited the strongest overall binding affinity for C5AR1 (-7.87 ± 0.83 kcal/mol), with key hydrogen bonds formed between C5AR1 and specific amino acid residues of the metabolite, further confirming the stability of this interaction.

**Table 1:** Molecular docking results of metabolites with core targets.

Protein	Metabolites	Binding energy (kcal/mol)	Grid box Center	Grid box dimension
C5AR1	1,3−Diphenylpropan−2−Ol	-6.9	51,45,43	size_x =19, size_y = 19, size_z = 19
		-8.4	126,3,5	size_x =22, size_y = 22, size_z = 22
		-8.3	111,-6,48	size_x =22, size_y = 22, size_z = 22
		-7.8	108,-1,35	size_x =22, size_y = 22, size_z = 22
		-7.7	104,-5,14	size_x =35, size_y = 35, size_z = 22
		-6.7	122,2,52	size_x =22, size_y = 22, size_z = 22
		-9.1	104,-5,14	size_x =35, size_y = 35, size_z = 22
		-9.0	111,-6,48	size_x =22, size_y = 22, size_z = 22
		-7.7	126,3,5	size_x =22, size_y = 22, size_z = 22
		-7.1	108,-1,35	size_x =22, size_y = 22, size_z = 22
HCAR3	3−Indolepropionic Acid	-7.2	140,121,90	size_x = 19, size_y = 19, size_z = 19
		-6.7	141,146,156	size_x = 26, size_y = 35, size_z = 32
		-5.8	127,115,161	size_x = 29, size_y = 31, size_z = 19
		-5.8	122,154,127	size_x = 27, size_y = 19, size_z = 19
		-5.6	120,96,167	size_x = 19, size_y = 19, size_z = 26
		-6.6	141,146,156	size_x = 26, size_y = 35, size_z = 32
		-5.9	122,154,137	size_x = 27, size_y = 19, size_z = 19
		-7.1	140,121,90	size_x = 19, size_y = 19, size_z = 19
		-7.3	140,121,90	size_x = 19, size_y = 19, size_z = 19
		-5.5	122,154,127	size_x = 27, size_y = 19, size_z = 19

The left panel of **Figure 8** displays the electrostatic surface potential distribution of the target protein, where red, blue, and gray regions represent negatively charged, positively charged, and neutral areas, respectively, providing an intuitive view of the charge distribution on the protein surface. The middle panel shows the three-dimensional spatial conformation of the target protein, depicted in blue. The right panel provides a magnified view of the ligand binding site, in which the metabolite structure is shown in light blue, yellow dashed lines indicate hydrogen bonds, and the light green region marks the binding interface between the protein and the metabolite.

These three genes were not only significantly enriched in IBD-related pathways (TNF, NF-κB, IL-17), but also closely associated with gut microbiota-derived metabolites (verified by molecular docking), indicating that they are key nodes connecting gut microbiota and IBD inflammation.

### 3.9 Evaluation of drugability and toxicity of metabolites

As summarized in **Table 2**, SwissADME analysis revealed that both 1,3-Diphenylpropan-2-Ol and 3-Indolepropionic Acid exhibited no Lipinski violations, with bioavailability scores of 0.85 and 0.55, respectively, indicating favorable oral absorption potential.

**Table 2:** Evaluation of physicochemical properties and drug-like properties of metabolites.

Compound	MW(g/mol)	HBA	HBD	MLog P	Lipinski’s violations	Bioavailability Score	TPSA
1,3−Diphenylpropan−2−Ol	189.21	2	2	1.40	No	0.85	53.09
3−Indolepropionic Acid	212.29	1	1	3.60	No	0.55	20.23

MW, molecular weight; HBA, hydrogen bond acceptors; HBD, hydrogen bond donors; MLogP, lipophilicity index; Lipinski violations, number of violations of Lipinski’s rule of five; Bioavailability score, oral bioavailability prediction score; TPSA, topological polar surface area.

Toxicity evaluation using ADMETlab 2.0 demonstrated that 1,3-Diphenylpropan-2-Ol showed no hERG inhibition, hepatotoxicity, carcinogenicity, or skin sensitization risks, suggesting a favorable safety profile (**Table 3**). 3-Indolepropionic Acid also showed no hERG inhibition, hepatotoxicity, or carcinogenicity, but presented a mild risk of skin sensitization. Overall, both metabolites meet basic druggability criteria, with only a minor skin sensitization concern for 3-Indolepropionic Acid, supporting their potential for further *in vivo* investigation and translational development.

**Table 3:** Toxicity assessment of core metabolites.

Compound	hERG blockers	H-HT	DILI	Carcinogenicity	Skin sensitization
1,3−Diphenylpropan−2−Ol	Non-blocker	negative	negative	negative	negative
3−Indolepropionic Acid	Non-blocker	negative	negative	negative	positive

hERG Blockers, human ether-à-go-go-related gene channel blockade risk; H-HT, hepatotoxicity; DILI, drug-induced liver injury; Carcinogenicity, carcinogenic potential; Skin Sensitization, skin sensitization risk.

## 4 Discussion

To address the limitation that existing IBD studies on the gut microbiota are confined to single methodologies or isolated pathways and lack a systematic “microbiota-metabolite-target” regulatory network with multi-method cross-validation, this study established a comprehensive regulatory axis in IBD by integrating transcriptome profiling, WGCNA, multi-database intersection, and three machine learning algorithms (LASSO, Boruta, and SVM-RFE). We identified C5AR1, KDM2A, and HCAR3 as core targets of gut microbiota-derived metabolites, thereby filling the gap in understanding the molecular mechanisms by which gut microbiota-derived metabolites regulate IBD pathogenesis.

Based on our findings, we propose a potential regulatory mechanism of the “gut microbiota-metabolite-target” axis in IBD: Gut microbiota dysbiosis leads to the production of metabolites such as 1,3-Diphenylpropan-2-Ol and 3-Indolepropionic acid, which directly bind to C5AR1 and KDM2A. Specifically, C5AR1 recruits immune cells to drive intestinal inflammation, KDM2A modulates the balance of inflammatory responses in the intestinal mucosa, and HCAR3 mediates crosstalk between microbial metabolites and host immunity. The TNF, NF-κB, and IL-17 signaling pathways may mediate the downstream effects of these targets, forming a complete “gut microbiota-metabolite-target-pathway-IBD” regulatory network.

Enrichment analysis revealed that the 19 candidate genes were significantly enriched in the TNF, NF-κB, and IL-17 signaling pathways. These findings are consistent with accumulating evidence that gut microbiota dysbiosis compromises intestinal barrier integrity, thereby facilitating the translocation of microbial metabolites and lipopolysaccharides into the intestinal mucosa and contributing to chronic intestinal inflammation and injury (Rolhion and Sokol, 2025; Xie et al., 2025). Mechanistically, the TNF signaling pathway, a master regulator of inflammation, has been extensively implicated in IBD pathogenesis, where it drives epithelial cell apoptosis, disrupts tight junctions, and perpetuates mucosal inflammation (Wu et al., 2024; Nicolò et al., 2025). Similarly, the NF-κB signaling pathway acts as a central mediator of inflammatory responses in IBD by regulating the expression of pro-inflammatory cytokines such as IL-6, IL-1β, and TNF-α, while also promoting immune cell activation and recruitment to the inflamed gut (Morrison et al., 2023; Mukherjee et al., 2024). The IL-17 signaling pathway, predominantly mediated by Th17 cells, plays a critical role in neutrophil recruitment, antimicrobial peptide production, and epithelial barrier dysfunction, further exacerbating chronic intestinal inflammation (Gomez-Bris et al., 2023). Collectively, these findings suggest that gut microbial metabolites may influence IBD progression by modulating the TNF, NF-κB, and IL-17 signaling pathways, thereby providing a mechanistic link between gut microbiota dysbiosis and IBD pathogenesis.

PPI network analysis combined with three machine learning algorithms (LASSO, Boruta, and SVM-RFE) identified C5AR1, KDM2A, and HCAR3 as core hub genes linking gut microbiota-derived metabolites to IBD pathogenesis. These genes participate in key biological processes related to intestinal inflammation and immune regulation. Specifically, C5AR1, as a complement receptor (enriched in MF term ‘complement receptor activity’), may recruit neutrophils and macrophages to the inflamed gut, thereby activating the TNF/NF-κB signaling pathway and promoting intestinal inflammation (Yu and Liu, 2023). Previous studies have reported that KDM2A regulates inflammatory gene expression and modulates the balance of pro-inflammatory and anti-inflammatory responses in the intestinal mucosa, potentially through epigenetic mechanisms (Xue et al., 2025; Li et al., 2026). HCAR3, as a metabolite-sensing receptor, mediates the crosstalk between gut microbiota metabolites and host immunity, maintaining intestinal homeostasis (Wnorowski et al., 2021; Spathakis et al., 2024). Collectively, these hub genes act as direct molecular targets of gut microbiota-derived metabolites and may modulate the TNF, NF-κB, and IL-17 signaling pathways in IBD. Therefore, C5AR1, KDM2A, and HCAR3 represent promising diagnostic biomarkers and therapeutic targets for microbiota-directed interventions in IBD.

1,3-Diphenylpropan-2-Ol, a gut microbiota-derived metabolite, directly targets C5AR1 with a binding energy of -7.87 ± 0.83 kcal/mol, suggesting its potential to modulate C5AR1-mediated chemotaxis and inflammatory responses in the intestinal mucosa. Similarly, 3-Indolepropionic acid, a tryptophan metabolite produced by gut microbiota that possesses anti-inflammatory, potent antioxidant, and radical-scavenging properties (Li et al., 2025; Owe-Larsson et al., 2025; Zhang et al., 2026), binds to HCAR3 with a binding energy of -6.35 ± 0.70 kcal/mol. This interaction may enhance the regulatory function of HCAR3, thereby maintaining intestinal homeostasis and alleviating intestinal inflammation in IBD. Collectively, these findings indicate that gut microbiota-derived metabolites may serve as potential therapeutic candidates in IBD by directly targeting the identified hub genes, thereby modulating key inflammatory pathways and restoring intestinal immune homeostasis.

Compared with previous studies on IBD and gut microbiota, this study has three key innovations: 1) It integrates three machine learning algorithms (LASSO, Boruta, SVM-RFE) for cross-validation, which overcomes the limitation of single-algorithm screening and improves the reliability of core hub gene identification; 2) It systematically constructs the ‘gut microbiota-metabolite-target’ regulatory axis, filling the gap of incomplete regulatory network in previous studies; 3) It verifies the direct binding between gut microbiota-derived metabolites and hub proteins by molecular docking, providing direct molecular evidence for the regulatory axis.

In terms of target screening, Abdelkader Oumeddour identified IBD-related genes using single WGCNA analysis (Oumeddour, 2025), while this study combined DEGs, gut microbiota-associated genes and WGCNA module genes, and further validated by three machine learning algorithms, making the core targets more reliable. Regarding metabolite validation, the gutMGene v2.0 database reported gut microbe-metabolite-host gene associations but did not verify the actual binding between metabolites and targets (Qi et al., 2025); in contrast, this study confirmed the stable binding of 1,3-Diphenylpropan-2-Ol to C5AR1 and 3-Indolepropionic Acid to HCAR3, providing a direct basis for metabolite-based intervention. With respect to clinical value, Wnorowski et al. proposed HCAR3 as a potential drug target for IBD but did not link it to gut microbiota-derived metabolites (Wnorowski et al., 2021), while this study clarified the upstream regulatory mechanism of HCAR3, expanding its application potential in microbiota-targeted therapy.

This study has several limitations that need to be addressed in future research. First, the sample size of the GEO datasets used in this study is moderate (GSE3365: 127 samples; GSE47908: 60 samples), which may affect the generalizability of the results. Future studies will collect fecal/serum samples from a larger cohort of IBD patients (n > 200) to verify the correlation between metabolite levels and hub gene expression and analyze their association with disease severity and prognosis. Second, IBD encompasses diverse clinical subtypes beyond the broad UC and CD classifications, including variations in disease location, behavior, and severity. However, the GEO datasets used in this study did not provide detailed clinical subclassification information, which prevented us from performing subtype-specific analyses. Future prospective studies with well-characterized clinical annotations are needed to investigate the associations between metabolite levels, hub gene expression, and specific IBD subtypes. Third, while our analysis suggests that gut microbiota-derived metabolites such as 1,3-Diphenylpropan-2-Ol and 3-Indolepropionic Acid play important roles in IBD, we did not identify the specific gut bacterial taxa possessing the biosynthetic genes for these metabolites, nor did we characterize how their populations change in the context of IBD. Future studies combining metagenomic sequencing with metabolomic profiling are needed to pinpoint the microbial producers and enzymes responsible for the production of these metabolites, and to investigate their abundance shifts in IBD patients. Fourth, the hub genes were identified based on transcriptomic data and machine learning models, and these results are computational predictions rather than functional evidence of causal roles in IBD pathogenesis. Fifth, molecular docking only verified the binding affinity, and future *in vitro* experiments (e.g., Western blot to detect the expression of C5AR1/KDM2A/HCAR3 in IBD cell models treated with metabolites, Transwell assay to detect immune cell migration) will verify the regulatory effect of metabolites on target genes; *in vivo* experiments using DSS-induced colitis mice will further validate the therapeutic efficacy of 1,3-Diphenylpropan-2-Ol and 3-Indolepropionic Acid. Sixth, the regulatory network was constructed based on database associations. Future studies will further explore the specific molecular mechanisms of the ‘gut microbiota-metabolite-target’ axis, such as the role of TNF/NF-κB/IL-17 signaling pathways in the regulatory process, and whether there is crosstalk between the three hub genes.

## 5 Conclusion

This study integrated multiple methods to construct the ‘gut microbiota-metabolite-hub gene’ regulatory axis in IBD for the first time. C5AR1, KDM2A, and HCAR3 were identified as core hub genes with computationally predicted diagnostic value, and 1,3-Diphenylpropan-2-Ol and 3-Indolepropionic Acid were computationally predicted to have strong binding affinity with C5AR1 and HCAR3, respectively. These findings provide novel candidate biomarkers and potential intervention molecules for IBD that merit further investigation and lay a preliminary theoretical basis for the development of microbiota-targeted precision therapy pending experimental corroboration. Specifically, C5AR1/KDM2A/HCAR3 can be used as potential therapeutic targets, and 1,3-Diphenylpropan-2-Ol/3-Indolepropionic Acid can be developed as candidate probiotic metabolites or dietary supplements for IBD intervention.

## Data Availability

The transcriptome datasets analyzed in this study are publicly available in the NCBI Gene Expression Omnibus (GEO) database under accession numbers GSE3365 (GPL96 platform) and GSE47908 (GPL570 platform). No new datasets were generated during the current study. All data used are publicly accessible and were analyzed in accordance with the GEO data usage policies. Further inquiries regarding data processing or analysis can be directed to the corresponding author.

## Supplementary material

Supplementary Figure 1 Batch-effect correction for the three hub genes across GPL96 and GPL570 platforms. **(A)** Box plots showing raw expression distributions of C5AR1, HCAR3, and KDM2A before ComBat correction. Notable batch-specific deviations are observed between the two platforms. **(B)** Box plots showing expression distributions after ComBat correction. The medians and interquartile ranges of all three genes are well aligned between platforms, confirming effective removal of batch effects.

Supplementary Figure 2 Sensitivity analysis of gene set selection strategies. **(A)** Bar plot of the top enriched GO terms for the WGCNA-only gene set (n = 280). **(B)** Circular plot of the corresponding GO enrichment results. The four concentric layers are organized as follows: the outermost layer displays GO functional IDs, categorized into three main ontologies (BP, CC, and MF); the second layer uses color intensity to indicate significance levels, with segment size and numerical values reflecting the number of genes enriched in each term; the third layer shows the counts of upregulated and downregulated genes within each term, distinguished by different colors; and the innermost layer consists of color blocks representing distinct functions, where block size indicates the Rich Factor (larger values denote greater enrichment).

Supplementary Figure 3 ROC analysis of established IBD biomarkers TNF and IL6. **(A)** Training cohort; **(B)** validation cohort. IL23R was excluded due to its lack of concurrent presence across the two datasets. Both markers showed AUC values below 0.7 in the training set but above 0.7 in the validation set, indicating inconsistent cross-cohort performance.

Supplementary Table 1 Complete list of gut microbiota-associated genes (n = 1,274) retrieved from the gutMGene database (v2.0) and used for candidate gene intersection analysis.

Supplementary Table 2 ROC performance metrics of the three hub genes (C5AR1, KDM2A, and HCAR3) in both the training and validation cohorts, including optimal cut-off values, sensitivity, specificity, and AUC values.

Supplementary Table 3 Full list of DEGs identified between IBD and control groups. DEGs, differentially expressed genes; IBD, inflammatory bowel disease.

Supplementary Table 4 Genes assigned to each WGCNA module.

Supplementary Table 5 Complete GO enrichment results for the 19 candidate genes, covering BP, CC, and MF terms with adjusted p-values < 0.05. GO, Gene Ontology; BP, biological process; CC, cellular component; MF, molecular function.

Supplementary Table 6 Complete KEGG pathway enrichment results for the 19 candidate genes. KEGG, Kyoto encyclopedia of genes and genomes.
