# Supplementary material for: Gut microbiota-derived metabolites target C5AR1/KDM2A/HCAR3 axis in inflammatory bowel disease: a multi-machine learning algorithms and molecular docking study
Source: Front Cell Infect Microbiol. 2026 Aug 21;16:1912373. doi: 10.3389/fcimb.2026.1912373 (PMC13541491; doi:10.3389/fcimb.2026.1912373)
Supplement: Supplementary file 1 [file Table1.docx]

Supplementary Table S1. ROC performance metrics of the three hub genes in training and validation sets.

| Gene | AUC | Opt_cutoff | Sensitivity | Specificity |
| --- | --- | --- | --- | --- |
| Train |  |  |  |  |
| C5AR1 | 0.878 | 8.66 | 0.706 | 0.929 |
| KDM2A | 0.905 | 7.543 | 0.8 | 0.929 |
| HCAR3 | 0.875 | 7.615 | 0.729 | 0.857 |
| Test |  |  |  |  |
| C5AR1 | 0.739 | 8.917 | 0.422 | 1 |
| KDM2A | 0.859 | 7.237 | 0.889 | 0.733 |
| HCAR3 | 0.852 | 6.886 | 0.756 | 0.933 |
